# Successful Laparoscopic Ureterolithotomy to Remove a Large Ureteral Stone in an Infant: A Case Report

**DOI:** 10.7759/cureus.83349

**Published:** 2025-05-02

**Authors:** Mohamad Khalid Al Aswad, Hussein Huwaijah, Mohammed Istarabadi

**Affiliations:** 1 College of Medicine, University of Sharjah, Sharjah, ARE; 2 Urology, NMC Royal Hospital Sharjah, Sharjah, ARE

**Keywords:** extracorporeal shock wave lithotripsy, hydronephrosis, individualized treatment planning, laparoscopic ureterolithotomy, pediatric urolithiasis, percutaneous nephrolithotomy, ureteroscopy, urinary tract infections

## Abstract

Pediatric urolithiasis, while rare, can result in significant morbidity; it typically presents with pain, irritability, and complications such as hydronephrosis and urinary tract infections. Conventional management techniques include extracorporeal shock wave lithotripsy (ESWL), ureteroscopy (URS), and percutaneous nephrolithotomy (PCNL), with the choice guided by stone size, location, and patient age. In select cases - particularly those involving large or impacted stones where traditional methods may be inadequate - laparoscopic ureterolithotomy can offer a safe and effective alternative. We present the case of a 21-month-old infant referred from another hospital for a large left ureteral stone. On examination, the patient was irritable and difficult to console but hemodynamically stable, with no other abnormal findings. A non-contrast CT scan confirmed a 1.5 × 0.7 × 0.8 cm stone in the upper left ureter, accompanied by mild hydronephrosis. Laparoscopic ureterolithotomy was performed successfully, resulting in complete stone clearance without postoperative complications. This case underscores the importance of individualized treatment planning in pediatric urolithiasis and highlights the efficacy of laparoscopy as a viable option in complex cases where standard approaches may not be feasible.

## Introduction

Pediatric urolithiasis refers to the presence of stones in the urinary tract of children, most commonly due to metabolic abnormalities such as hypercalciuria, hypocitraturia, and hyperoxaluria. Symptoms vary with age but often include hematuria, abdominal or flank pain, urinary tract infections, and irritability in younger children [1]. The prevalence of pediatric urolithiasis is on the rise, with one study reporting a peak of 65.2 cases per 100,000 person-years in 2011 and another showing a 25% increase in incidence every five years among adolescents between 1997 and 2012 [2,3]. In recent years, there has been a notable evolution in the management of kidney and ureteral stones in the pediatric population, particularly with the adoption of minimally invasive techniques.

As emphasized by Paraboschi et al. [4], there is a measurable trend of shift towards the treatment of pediatric urolithiasis with less invasive procedures, using miniaturized instruments. This is especially significant in children, where there is a true risk of complications from invasive surgeries. Although there are various treatment options for such cases, including conservative management, extracorporeal shock wave lithotripsy (ESWL), and ureteroscopy (URS), the laparoscopic approach is gaining significant attention as a safe and effective option [4,5]. This is especially applicable when other endoscopic approaches are not feasible due to abnormal renal anatomy or a large stone size [6,7]. Such an approach is associated with several advantages, including the smaller incision size, the reduction in postoperative pain, and a faster recovery time [4,5]. This case report highlights the successful management of a 21-month-old infant who presented with a large left ureteric stone, via a laparoscopic approach.

## Case presentation

A 21-month-old male child, delivered at full term without complications, was referred to our tertiary hospital in Sharjah, United Arab Emirates, due to a chief complaint of left flank pain and irritability; he was diagnosed with pediatric nephrolithiasis. He had no notable medical, surgical, or urological history and had maintained good health before this presentation. Upon initial evaluation, the patient's vital signs were normal. The physical examination revealed no abnormalities. The patient showed irritability and was difficult to console, possibly due to the presence of stones; nevertheless, there were no signs of infection.

**Figure 1:**
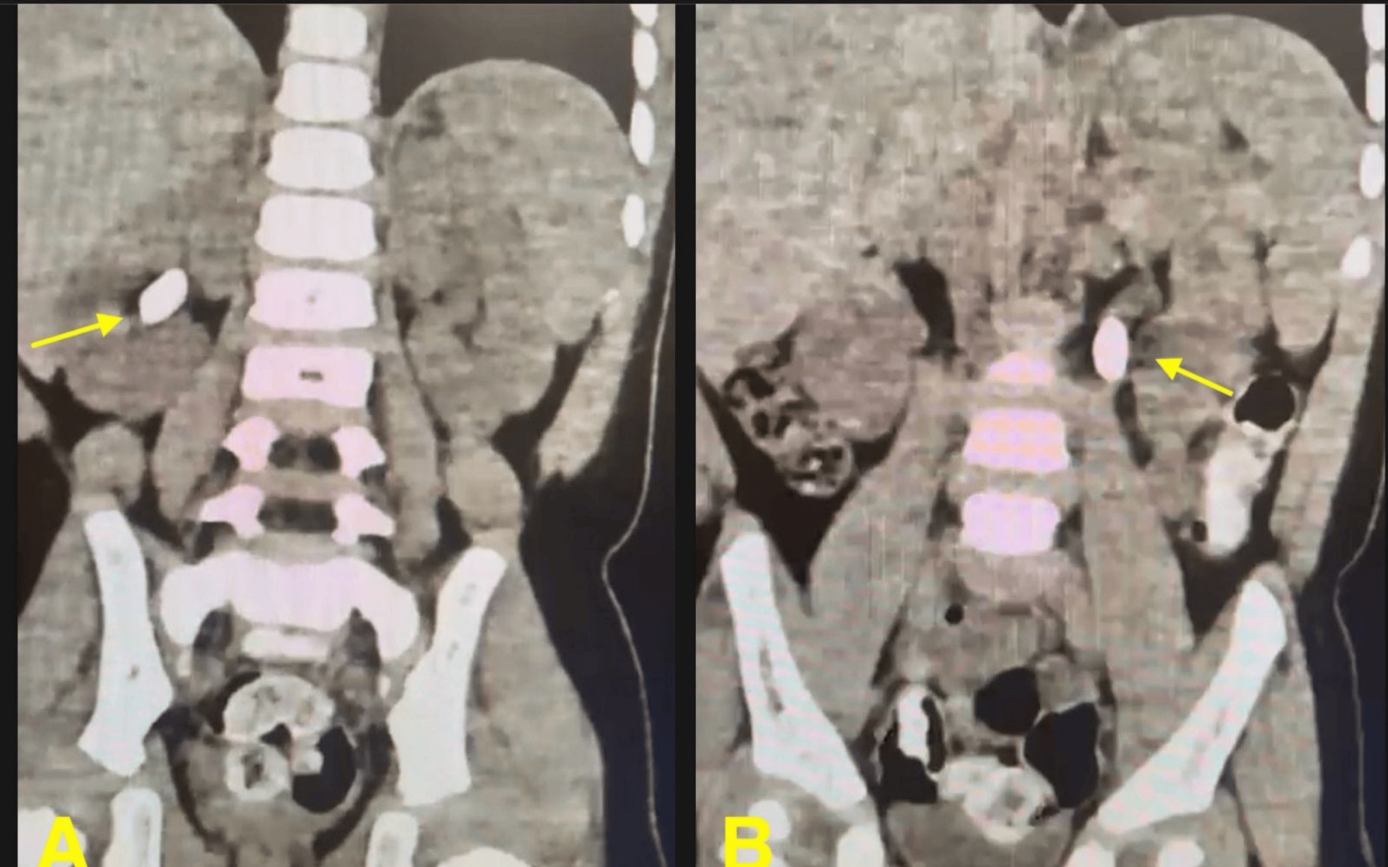
Coronal CT images demonstrating bilateral urolithiasis (A) Right renal stone (yellow arrow) located within the renal pelvis. (B) Left ureteral stone (yellow arrow) positioned within the ureter, causing partial obstruction CT: computed tomography

Complete investigations were conducted, including a full blood count and electrolyte profile (Table 1). The findings were mostly normal, except for slightly elevated calcium levels. This finding was clinically relevant, as hypercalcemia is a known risk factor for calcium-based nephrolithiasis. The serum creatinine concentration was 0.29 mg/dL, indicating normal renal function. A helical kidneys, ureters, and bladder (KUB) CT scan identified a substantial left ureteric stone measuring 1.5 x 0.7 x 0.8 cm, as well as mild proximal hydronephrosis. The stone was located in the proximal portion of the left ureter. A right staghorn renal stone measuring 1.5 x 0.7 x 1.0 cm was found with no associated hydronephrosis (Figure 1).

**Table 1:** Laboratory findings HCT: hematocrit; MCH: mean corpuscular hemoglobin; MCHC: mean corpuscular hemoglobin concentration; MCV: mean corpuscular volume; MPV: mean platelet volume; RBC: red blood cells; RDW-CV: red cell distribution width - coefficient of variation; WBC: white blood cells

Test	Result	Units	Reference range
WBC	12.8	x10^9^/L	6.0 - 17.5
RBC	4.8	x10^12^/L	3.8 - 4.8
Hemoglobin	11.8	g/dL	10.5 - 13.5
HCT	37.6	%	33 - 49
MCV	79.0	fL	70 - 86
RDW-CV	12.8	%	11.5 - 16.0
Platelets	395.0	x10^9^/L	150 - 450
MCH	24.8	pg	22 - 31
MCHC	31.4	g/dL	30 - 36
MPV	9.5	fL	9.6 - 12.0
Neutrophils	54.9	%	15 - 35
Lymphocytes	30.7	%	45 - 76
Monocytes	10.8	%	3 - 6
Eosinophils	2.9	%	0 - 3
Basophils	0.7	%	0 - 2
Potassium, serum	4.2	mmol/L	3.5 - 5.1
Bicarbonate, serum	24.2	mmol/L	20 - 31
Sodium, serum	136.0	mmol/L	136 - 145
Chloride, serum	104.0	mmol/L	98 - 107
Blood urea, serum	25.68	mg/dL	7.2 - 36
Creatinine, serum	0.29	mg/dL	0.1 - 0.4

**Figure 2:**
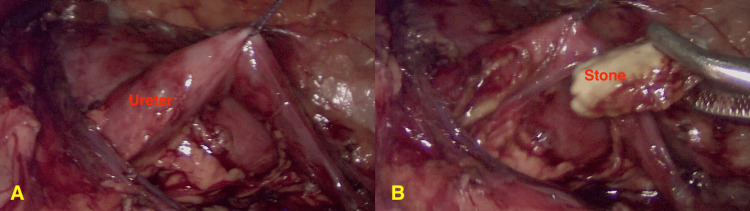
Intraoperative images demonstrating the surgical approach for ureterolithotomy (A) The ureter is carefully mobilized and fixated to the peritoneum to optimize exposure and facilitate stone removal. (B) Extraction of the ureteral stone using surgical forceps following ureterotomy

Given the size and the location of the left stone, along with the patient's age, a laparoscopic approach was determined to be the most appropriate option. Although ESWL and URS are generally considered first-line treatments for proximal ureteral stones, the stone in this case was impacted, and the patient’s very young age made ureteroscopic access technically challenging, justifying the choice of a primary laparoscopic approach in line with guideline-based exceptions. The procedure was conducted under general anesthesia, utilizing three 5-mm ports - one for the laparoscope and two for surgical instruments. The peritoneum was incised and reflected over the descending colon to reach the retroperitoneal area, allowing visualization of the left ureter. Following the localization of the stone, a stay stitch was positioned distal to the stone for retraction of the ureter. The ureter was incised longitudinally, and the stone was extracted from the ureter and retrieved through the port, and a 4.3 Fr ureteric stent was inserted into the ureter. The ureter gap was then sutured using a 5.0 absorbable suture (Figure 2). A drain was placed, and the stone was sent for analysis, which revealed its composition to be 60% calcium oxalate monohydrate, 30% calcium oxalate dihydrate, and 10% carbonate apatite.

The surgical procedure went smoothly without any intraoperative complications, achieving 100% stone clearance. The patient was closely monitored and started on oral fluids and soft foods on the first postoperative day. Pain management was mostly achieved with 1000 mg acetaminophen, and the patient showed significant improvement within 48 hours. The drain was removed without any issues. Furthermore, there were no postoperative complications like hemorrhage or infection. The patient was discharged on the third postoperative day and scheduled for a follow-up in two weeks. The right staghorn renal stone was scheduled for removal at a later date.

## Discussion

The management of pediatric nephrolithiasis requires a thorough evaluation of treatment options, taking into account the patient's age, stone features, and possible risks associated with various procedures. The laparoscopic method was considered the most appropriate in our case, as alternative treatment options were unsuitable [7]. Conservative management was considered inadequate due to the stone's size and the potential for blockage. Percutaneous nephrolithotomy (PCNL) poses higher risks in young patients, such as bleeding and organ injury [6]. Open surgery is reserved for instances with anatomical defects, as it is linked to greater morbidity and prolonged recovery. URS presents problems for young children due to the small ureteral diameters, increasing the risk of injury to the ureter and increasing the risk of vesicoureteric reflux.

A study by Ishii et al. [8] indicated an elevated complication and failure rate in pediatric patients, particularly those under six years of age, when managed with URS. Although beneficial in specific circumstances, ESWL may result in stone fragmentation, leading to symptomatic episodes of renal colic, hematuria, and urinary tract infection, which can be bothersome to a pediatric patient [9]. Laparoscopic surgery provides reduced postoperative pain, shorter hospitalizations, and faster recovery, making it an optimal choice for efficient stone removal with reduced risk [10]. This is further supported by the findings of Srivastava et al. [11], who reported a 100% stone-free rate and minimal complications in a large pediatric cohort undergoing laparoscopic ureterolithotomy, particularly for impacted ureteral stones ≥1.5 cm.

Laparoscopic procedures, however minimally invasive, carry potential risks such as infection and bleeding [12]. Landa-Juárez et al. [13] found complications in a subset of pediatric patients, with 12.5% needing blood transfusions and 18.7% encountering self-limited urine leaks. Urine leakage and subsequent growth of urinoma may arise when urine accumulates outside the urinary system. Additionally, though rare, omental prolapse - where the omentum protrudes through the surgical incision - has been documented, as reported by Agrawal et al. [14]. In our case, a precise surgical technique was utilized to reduce such risks. This involved the use of absorbable sutures with minimal bites to maintain the local blood flow and mitigate the danger of stricture formation resulting from scar tissue. The lack of postoperative problems in our patient shows the efficacy of this careful approach in preventing stricture formation.

Intraoperative challenges during pediatric laparoscopic ureterolithotomy include the risk of ureteral stricture, often due to ischemia from excessive dissection or tight suturing. Raheem et al. [15] recommend preserving periureteric blood supply through limited dissection, using fine absorbable sutures in a tension-free closure, and placing a JJ stent to support healing and prevent complications. Similarly, Yasui et al. [16] reported no strictures in their series when meticulous technique was used, reinforcing the importance of gentle handling, precise suturing, and stenting to minimize postoperative risks.

## Conclusions

This report demonstrates a unique approach for managing a large ureteral stone in a 21-month-old infant by laparoscopic ureterolithotomy. In cases where stone measurements, location, or patient anatomy restrict the efficacy or safety of traditional methods like ESWL, URS, or PCNL, laparoscopy presents a viable, minimally invasive option with advantageous results. The total stone removal and lack of perioperative problems in this instance highlight the procedure's safety and effectiveness when executed with precise surgical skill.
